# Supplementary material for: Higher interglacial dust fluxes relative to glacial periods in southwestern North American deserts
Source: Nat Commun. 2025 Nov 28;16:10718. doi: 10.1038/s41467-025-65744-6 (PMC12663173; doi:10.1038/s41467-025-65744-6)
Supplement: Supplementary file 1 — Supplementary Information [file 41467_2025_65744_MOESM1_ESM.pdf]

Higher interglacial dust fluxes relative to glacial periods in southwestern North America deserts

Supplementary Information

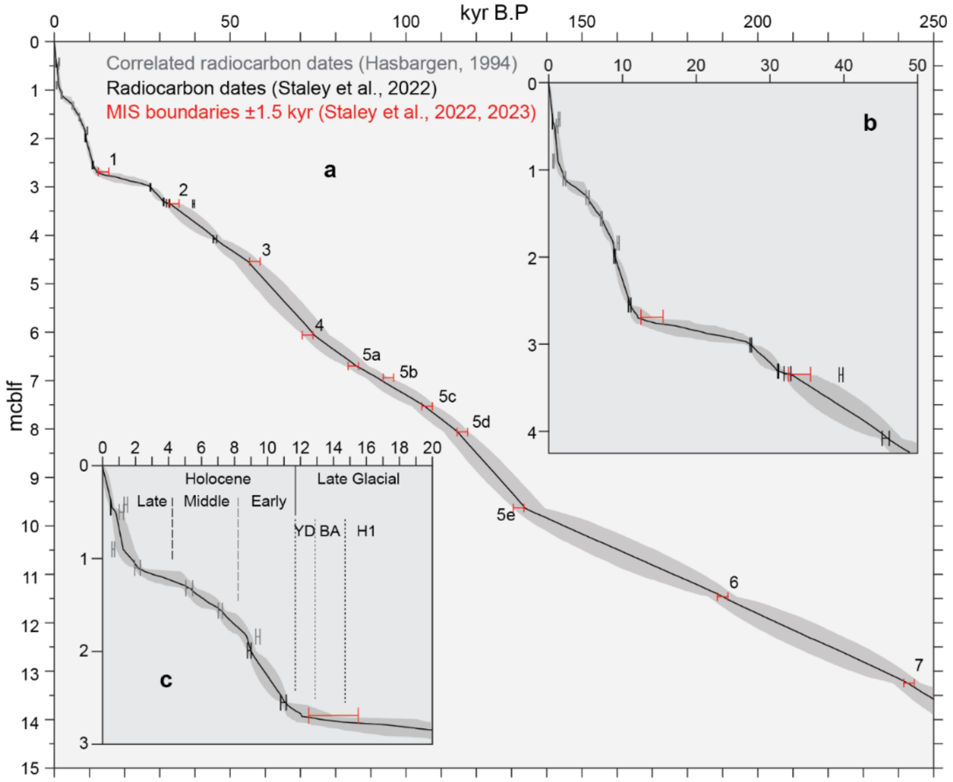

Supplementary Figure 1 | STL14 age model Bayesian age model generated using Bacon (v.2.3) software[1,2]. Input dates include radiocarbon dates from core STL14[3] (black), correlated radiocarbon stratigraphy from another Stoneman Lake sediment core[2,4] (gray), and correlation of facies changes to global climate transitions[2,3] (red). **a**, age model for top 14 meters. Inset **b**, the radiocarbon interval. Inset **c**, the last 20 kyr. MIS = Marine Isotope Stage; YD = Younger Dryas; BA = Bølling-Allerød; H1 = Heinrich Stadial 1.

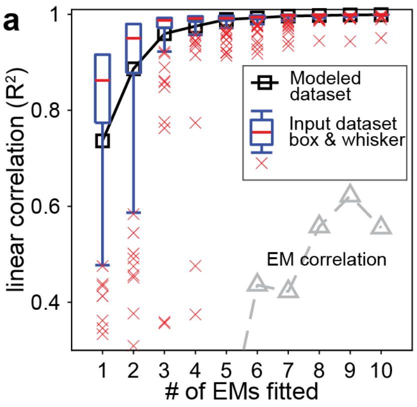

b

# of end members fitted	Accounts for: % of data set variance	% of specimen median variance	% of specimen lower 95%	angular deviation	median angular deviation	upper 95% angular deviation	maximum end member R^2
1	73.6	86	47.7	23.3	16.5	35.1	NaN
2	88.7	95	58.7	15.1	9.6	27.9	0.054
3	95.9	98.7	92.2	8.9	5.1	13.3	0.024
4	97.6	99.2	95.7	6.9	3.8	9.1	0.123
5	98.9	99.2	97.2	4.6	3.8	7.8	0.139
6	99.3	99.4	97.9	3.8	3.3	6.3	0.436
7	99.6	99.8	98.7	2.7	1.9	4.8	0.422
8	99.7	99.9	99.2	2.2	1.6	3.7	0.543
9	99.9	99.9	99.5	1.7	1.3	2.9	0.613
10	99.9	99.9	99.7	1.4	1	2.3	0.571

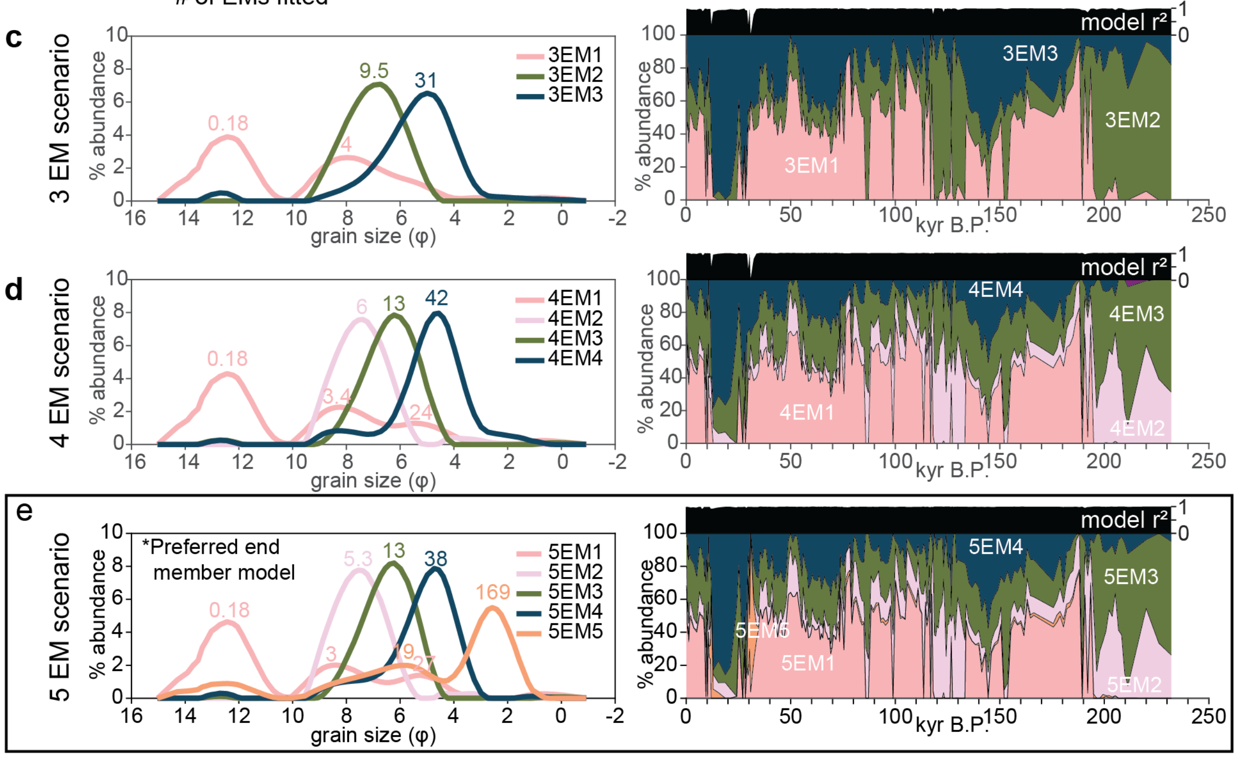

Supplementary Figure 2 | End member (EM) model scenarios: statistics and comparison of leading models **a**, linear correlation between the dataset constructed from fitted non-parametric end members in 1 to 10 end member scenarios and measured data from laser particle size analysis of core STL14 sediments. EM correlation is the maximum squared linear correlation between all fitted end members (last column in **b**). Overfitting occurs if the correlation is too high, e.g., the 6–10 end-member scenarios. Scenarios with fewer end members should be preferred[5]. **b**, model quality for each scenario, with the most likely models (3, 4, and 5 end-member scenarios) shaded in gray and the preferred model boxed. **c**, model output for the 3 end-member scenario. On the left are the particle size distributions of each end member. On the right, the top panel shows the model fit by depth (white intervals indicate suboptimal fit), while the bottom panel shows the modeled percentage of each end member through time. **d** and **e**, show the same information as **c** but for the 4 and 5 end-member scenarios with **e** being the preferred model.

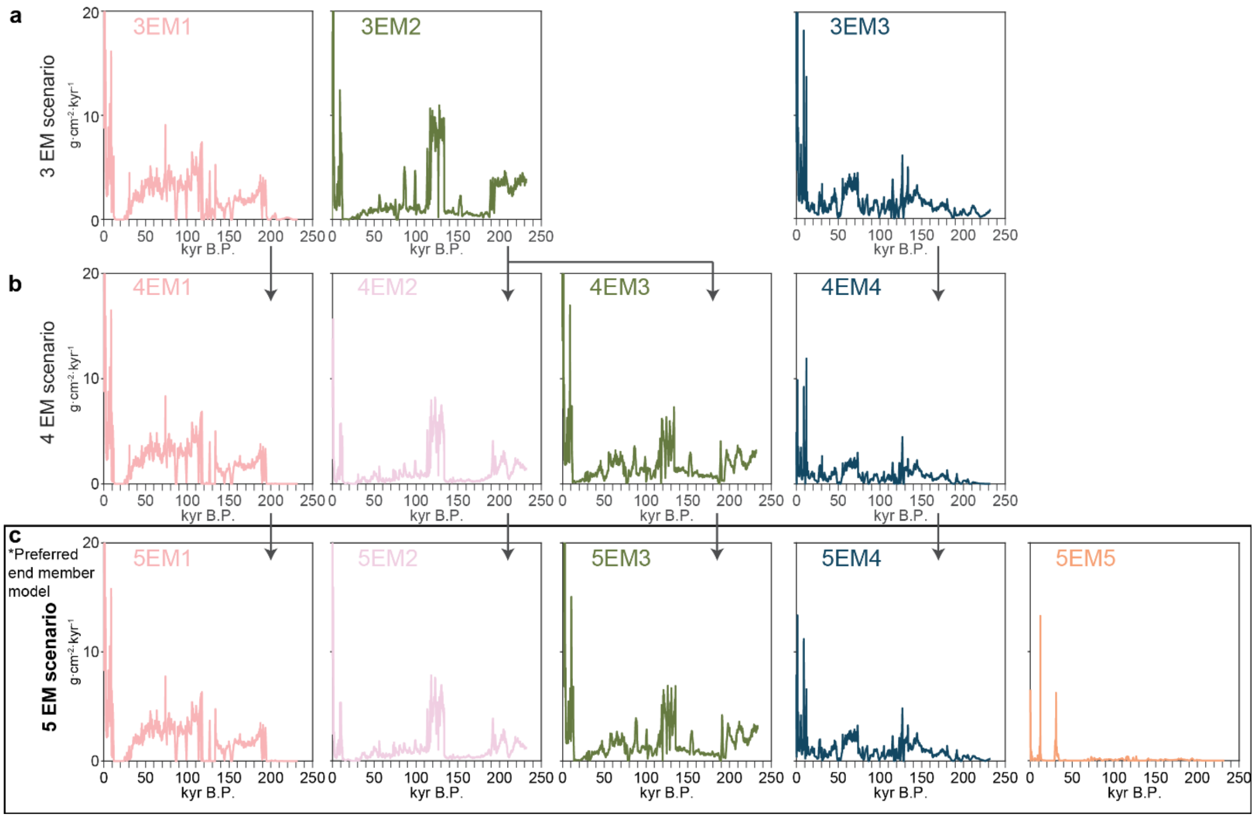

Supplementary Figure 3 | End member (EM) accumulation rates in the leading model scenarios End member accumulation rates were calculated by multiplying the sedimentation rate, clastic dry density, and percent of clastic volume accounted for by each end member (see text for details on this calculation). **a**, **b**, and **c** show the results for the 3, 4, and 5 end-member scenarios, respectively. Arrows indicate relationships between end members in the different scenarios.

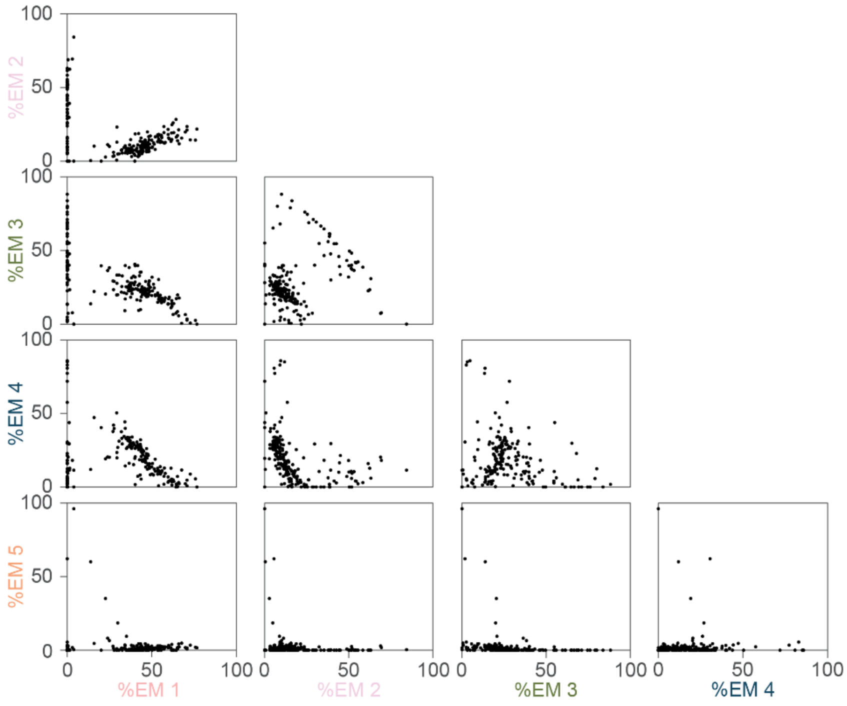

Supplementary Figure 4 | End-member (EM) abundance correlation in the 5EM scenario Correlation plots of end-member percentages. Slight correlation between EM1 and EM2 supports the interpretation they both represent catchment derived clastic sediment populations. Weak to negative correlations between EM1 and EM2 compared to EM3 and EM4 support the interpretation they come from different sources.

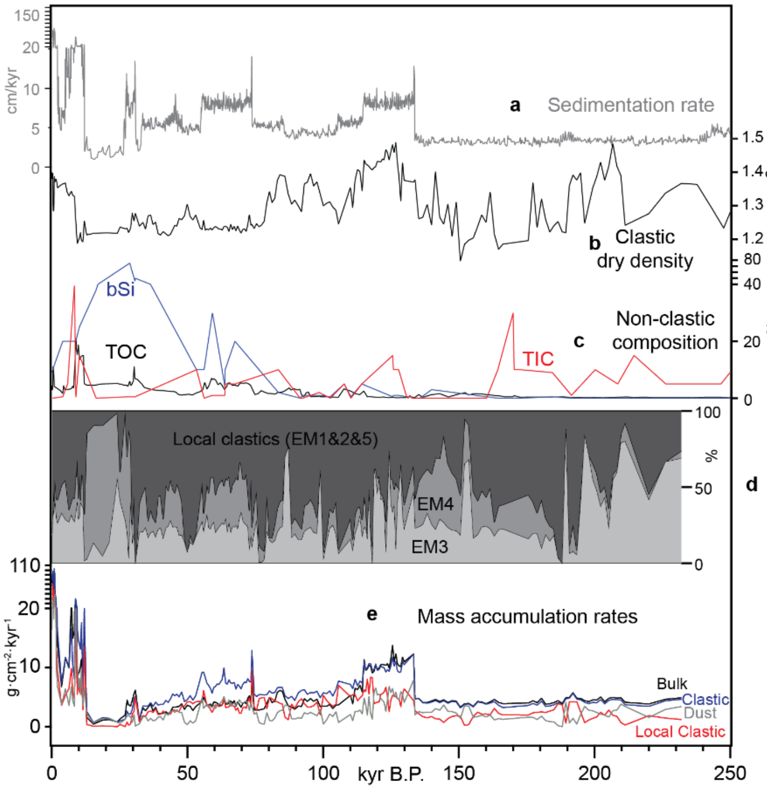

Supplementary Figure 5 | Data used to calculate mass accumulation rates

a, sedimentation rate based on the median age model (Supplementary Fig. 1). **b**, clastic dry density (derived from dry bulk density, Supplementary Fig. 6). **c**, non-clastic content (biogenic silica (bSi), organic matter (TOC), and inorganic carbon (TIC) in core STL14[3]. **d**, percentage of clastics modeled as local versus dust. **e**, calculated mass accumulation rates for bulk sediment, clastics, dust, and local clastics. Dust values presented here do not account for sediment focusing.

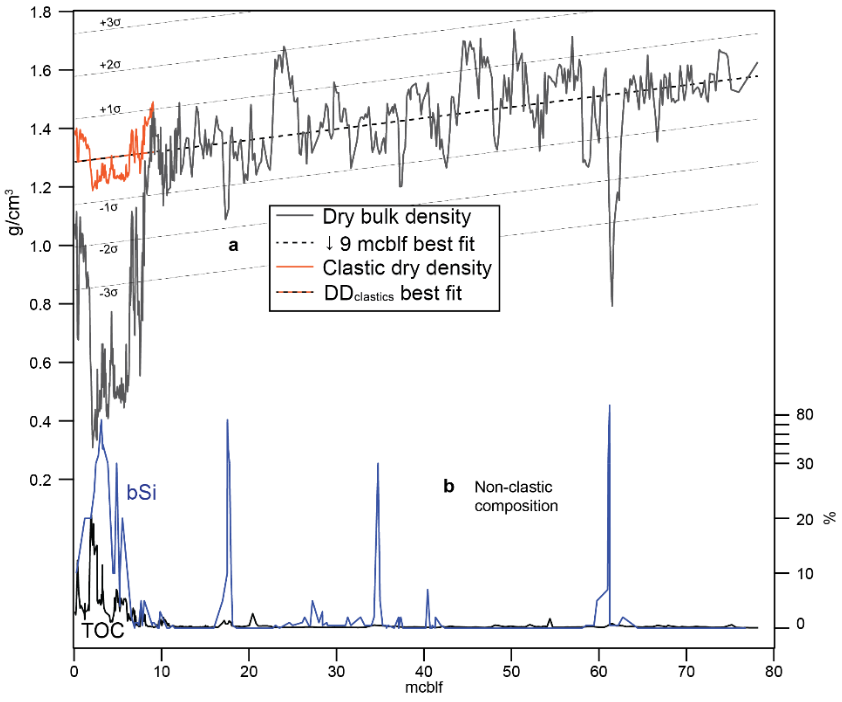

Supplementary Figure 6 | Adjusting dry bulk density to reflect the clastic dry density

a, dry bulk density[3] (gray line) with standard deviation (fine dashed lines parallel to the linear best fit (thick dashed line) and adjusted values (orange line) representing only the clastic dry density ($DD_{clastics}$). The adjusted linear best fit is shown with a dashed orange and black line. **b**, biogenic silica (bSi) and total organic carbon (TOC) in core sediments, highlighting the influence of non-clastic material on dry bulk density. mcblf = meters composite below lake floor.

References

1 Blaauw, M. & Christen, J. A. Bacon manual – v2.3.9.1. 15 (2011).

2 Staley, S. E. *et al*. Long-Term Landscape Evolution in Response to Climate Change, Ecosystem Dynamics, and Fire in a Basaltic Catchment on the Colorado Plateau. *Journal of Geophysical Research: Earth Surface* **128**, 1–19 (2023). https://doi.org/10.1029/2023JF007266

3 Staley, S. E., Fawcett, P. J., Anderson, R. S. & Jiménez-Moreno, G. Early Pleistocene–to–present paleoclimate archive for the American Southwest from Stoneman Lake, Arizona, USA. *Geological Society of America Bulletin* **134**, 791–814 (2022). https://doi.org/10.1130/b36038.1

4 Hasbargen, J. A Holocene Paleoclimatic and Environmental Record from Stoneman Lake, Arizona. *Quaternary Research* **42**, 188–196 (1994).

5 Paterson, G. A. & Heslop, D. New methods for unmixing sediment grain size data. *Geochemistry, Geophysics, Geosystems* **16**, 4494–4506 (2015). https://doi.org/10.1002/2015gc006070
